# Molecular characterization of *Neospora caninum* major antigens NcSAG1 and NcSRS2

**DOI:** 10.1098/rsos.250239

**Published:** 2025-08-20

**Authors:** Soledad Echeverría, Federico Carrión, Martín Soñora, Andrés Cabrera, Carlos Robello

**Affiliations:** ^1^ Laboratorio de Interacciones Hospedero Patogeno, Institut Pasteur de Montevideo, Montevideo, Uruguay; ^2^ Laboratorio de Inmunovirología / Unidad de Biofísica de Proteínas, Institut Pasteur de Montevideo, Montevideo, Uruguay; ^3^ Laboratorio de Simulaciones Biomoleculares, Institut Pasteur de Montevideo, Montevideo, Uruguay; ^4^ Unidad Académica de Parasitología, Facultad de Medicina, UDELAR, Uruguay; ^5^ Unidad Académica de Bioquímica, Facultad de Medicina, UDELAR, Uruguay

**Keywords:** SAG1-related sequence proteins, SRS, NcSAG1, NcSRS2, *Neospora caninum*, apicomplexa

## Abstract

The SAG1-related sequence (SRS) protein family was initially identified in *Toxoplasma gondii* as glycosyl-phosphatidylinositol-anchored surface antigens. More recently, they have been identified in *Neospora caninum*, the causative agent of neosporosis, a leading cause of bovine abortion worldwide. These proteins are implicated in parasite adhesion to and invasion of host cells, immune response modulation and structural roles in the cyst wall. In this study, we characterized two key *N. caninum* SRS proteins, NcSAG1 and NcSRS2, through sequence analysis, structural modelling, biophysical characterization and immunochemical assessment. Sequence analyses revealed conserved domains, including hallmark D1 and D2 regions, but with significant sequence divergence. Using AlphaFold, we constructed reliable structural models, confirming conserved features such as disulfide bond patterns and dimerization. Structural comparisons demonstrated a high degree of conservation within D1 domains despite low sequence similarity. Recombinant NcSAG1 and NcSRS2 were expressed as soluble and stable proteins in *Drosophila melanogaster* S2 cells, achieving yields comparable to the most efficient prokaryote expression systems. Size exclusion chromatography and dynamic light scattering demonstrated their dimeric nature and structural stability, with melting temperatures exceeding 50°C. Circular dichroism spectroscopy confirmed their correct secondary structure content, validating proper folding and structural integrity. Antigenicity assays demonstrated universal recognition by sera from experimentally and naturally infected hosts, highlighting their potential as diagnostic markers or vaccine candidates. Comparative structural analysis of 219 SRS family members, based on sequence and AlphaFold-predicted structures, revealed conserved cysteine, proline and tryptophan motifs. Hierarchical clustering and phylogenetic analyses identified key evolutionary clusters, correlating structural divergence with functional specialization. Discrepancies between sequence- and structure-based trees underscored instances of structural evolution not reflected in sequence data. This comprehensive analysis bridges sequence divergence, structural conservation and biological function, providing a robust framework for investigating SRS proteins’ roles in pathogenesis and immunity. Our findings lay the groundwork for future research into *N. caninum*’s molecular mechanisms and their implications for controlling neosporosis.

## 1. Introduction

The apicomplexan *Neospora caninum* is an obligate intracellular parasite closely related to *Toxoplasma gondii* [1,2]. Dogs act as the definitive hosts in the life cycle of *N. caninum*, where the sexual replication of the parasite occurs in the intestinal epithelium, culminating in the production of oocysts [3]. These oocysts are shed in the faeces of infected dogs, contaminating the environment and serving as a source of infection for both other canids and a wide range of herbivorous intermediate hosts, such as cattle and sheep [4,5]. In some cases, *N. caninum* infection in dogs can disseminate to the central nervous system, leading to chronic neurological manifestations, including ataxia, hind limb paresis and muscle atrophy [6]. The economic impact of neosporosis is most significant in cattle, where it causes bovine neosporosis [7], the leading cause of bovine abortion worldwide [8]. Infected cattle can abort from three months of pregnancy onwards, or the parasite can be transmitted vertically from mother to offspring [3]. Congenitally infected calves may or may not exhibit symptoms but can serve as carriers, maintaining the disease within the herd [3]. Various strategies have been proposed for controlling and diagnosing neosporosis, with surface antigen proteins showing high potential for diagnosis and vaccine development [9].

The SAG1-related sequence (SRS) protein family was initially identified in *T. gondii* as major surface antigens and named based on their apparent molecular weight (MW) in sodium dodecyl sulfate–polyacrylamide gel electrophoresis (SDS-PAGE) [10]. In 1991, a nomenclature was proposed using capital letters (e.g. P30 was called SAG1, and P22 was called SAG2) [11]. The SRS superfamily is also present in *N. caninum*, where orthologues for SRS proteins described in *T. gondii* were found [12]. Recent studies involving the genomes of both *T. gondii* and *N. caninum* revealed that the SRS family appears more extensive in *N. caninum* than in *T. gondii* [13,14]. SRS proteins exhibit a sequence identity of approximately 25%–30% and share several common characteristics, including an N-terminal signal peptide with 8–12 conserved cysteine residues, four conserved prolines, a conserved tryptophan in the membrane-proximal domain and a C-terminal glycosyl-phosphatidylinositol (GPI) signal. Most of them are anchored to the membrane by GPI (GPI-anchored) [15,16]. The crystal structures of SAG1, BSR4 and SporoSAG from *T. gondii* have been obtained [17–19]. These structures—crystallized without the signal peptide and the GPI anchor region—revealed the presence of two domains, D1 and D2, connected by a flexible hinge linker. These domains form a β-sandwich structure consisting of antiparallel and parallel strands stabilized by three conserved disulfide bonds. The reported structure appears as a homodimer, with the dimeric form resembling an immunoglobulin domain, suggesting a potential relationship to its proposed function in ligand recognition. This implies that the dimer would play a significant role in the protein’s ability to recognize and bind to specific ligands [19].

Studies on the tachyzoite-to-bradyzoite transition have revealed significant changes in the expression profiles of SRS proteins, probably reflecting their distinct roles in the parasite’s life cycle [15,20]. These expression patterns also vary among strains, correlating with infectivity and virulence: less virulent strains typically express a broader range of SRS proteins, including those associated with bradyzoites [21–23]. In *T. gondii,* the attachment to the host cell is primarily mediated by SAG1; although it is not the only interaction, it is important for adhesion and invasion [24]. In the case of *N. caninum*, two SRSs have been described for host cell attachment: NcSAG1 (also known as Ncp36) and NcSRS2 (also known as Ncp43 or Ncp35), playing essential roles in the invasion process [25–29]. The work of Nishikawa *et al*. [27] shows the relevance of NcSAG1 and NcSRS2 in the invasion process; by using monoclonal antibodies, the capacity of parasites to invade the host cell decreases significantly [27]. The role of NcSAG1 in the pathogenesis of *N. caninum* has been demonstrated by gene deletion studies, which revealed that knockout of NcSAG1 significantly reduces parasite infectivity and egress, in murine models [30]. The importance of NcSRS2 in the attachment and invasion was demonstrated using polyclonal and monoclonal antibodies for NcSRS2 and showing the decrease of attachment and invasion of *N. caninum* [28]. It was demonstrated that Nc-p43 (NcSRS2) is localized not only on the tachyzoite surface but also within the rhoptries and dense granules, suggesting that this protein is secreted [31].

Furthermore, its expression was observed in both tachyzoites and bradyzoites. NcSAG1 and NcSRS2 have been widely used in the development of ELISA-based diagnostic assays, as well as in vaccine research [32,33]. Despite extensive studies, the function, structure and physicochemical properties of SRS proteins remain poorly understood, including the nature of ligands they recognize on target cell surfaces and how their diversity influences host range. In this study, we expressed NcSRS2 and NcSAG1 in a eukaryotic system and performed biochemical and structural characterization of these proteins. Our findings provide helpful insights that could enhance the understanding of these proteins.

## 2. Material and methods

### 2.1. Cloning, protein expression and purification

Based on predictions from the SignalP algorithm, we excluded the signal peptide to produce the recombinant proteins. DNA sequences coding for amino acids 31−317 of *NcSAG1* (GenBank: AF123660) and 54−401 of *NcSRS2* (GenBank: AF061249), both fused to a C-terminal enterokinase cleavage site followed by a Twin-Strep-tag® (IBA) and a stop codon, were optimized for *Drosophila melanogaster* codon usage and synthesized by GenScript. Synthetic genes were cloned into a pMT/V5-His expression vector (Invitrogen) immediately after the BiP sequence as previously described [34].

S2 stable cell lines expressing rNcSAG1 and rNcSRS2 were obtained by co-transfection with the respective expression vector and pCoPuro selection plasmid using a 2 : 1 ratio, as previously described [34]. Transfections were achieved with an ExPERT STx transfection system using OC-400 processing assemblies (MaxCyte). Transfected cells were selected at 28°C in Insect Xpress medium (LONZA), added with 6 µg ml^−1^ puromycin. Stable S2 cell lines were grown in glass flasks at 28°C with 110 rpm agitation in a standard orbital shaker and induced at 5 × 10^6^ cells ml^−1^ with 5 µM CdCl_2_. After 3 days post-induction, cells were harvested by centrifugation at 150 g for 5 min, and both proteins were purified from the culture supernatant by affinity chromatography (AC) as before [34]. Briefly, culture supernatants were centrifuged at 6000*g*, loaded in 5 ml Strep-Tactin®XT 4Flow® columns (IBA), and eluted with 10 ml of 1 × Buffer BXT (Strep‑Tactin®XT elution buffer containing 50 mM biotin; IBA), following the manufacturer’s recommendations. An extra purification step by size exclusion chromatography (SEC) in 0.1 M Tris-Cl pH 8.0, 0.15 M NaCl and 1 mM EDTA (storage buffer) was conducted using Superdex 200 Increase 10/300 GL or HiLoad Superdex 200 16/600 pg columns (Cytiva). When required, the purification tag was removed by incubating SEC-purified proteins overnight at 25°C with Enterokinase-His (Genscript) (20 U mg^−1^) and subjected to a second AC step in the storage buffer. Cleaved proteins (rNcSRS2^*^ and rNcSAG1^*^) were collected in the flow-through fraction and subjected to SEC under the same conditions.

### 2.2. Analytical size exclusion chromatography

A volume of 100 μl was injected into a Superdex 200 Increase 10/300 GL column equilibrated in a storage buffer and eluted at 0.5 ml min^-1^. Under the same conditions, calibration was performed using low molecular weight (LMW) and high molecular weight (HMW) gel filtration calibration kits (Cytiva).

### 2.3. Nano-scale differential scanning fluorimetry

The thermostability of rNcSAG1-ST and rNcSRS2-ST was analysed by nano-scale differential scanning fluorimetry (nanoDSF) using a Prometheus NT.48 (Nanotemper). Unfolding and refolding curves were obtained by measuring intrinsic emission fluorescence at 350 and 330 nm upon sequential heating and cooling at 1°C min^−1^ between 20°C and 90°C, and melting temperature (*T*_M_) values were calculated from inflection points by following the first derivative of the *F*_350_/*F*_330_ ratio.

### 2.4. Dynamic light scattering

The hydrodynamic radius (R_H_) of recombinant proteins was analysed by dynamic light scattering (DLS) in a Zetasizer Nano S (Malvern) using low-volume quartz cuvettes (ZEN2112) at 25°C. Measurements were conducted in triplicate with rNcSAG1^*^ and rNcSRS2^*^ after samples were concentrated to 1 mg ml^−1^ by centrifugal ultrafiltration using Amicon® Ultra-15 tubes (Millipore).

### 2.5. Far-UV circular dichroism

Secondary structure analysis of rNcSAG1* and rNcSRS2* was achieved using a Chirascan V100 (Applied Photophysics) at 25°C. After Strep-tag removal, followed by protein ultrafiltration, sample absorbance at 280 nm was measured using a 10 mm pathlength quartz cuvette to calculate protein concentration. To obtain far-UV circular dichroism (far-UV CD) spectra, samples were diluted 1 : 20 in deuterium oxide to 3.04 and 3.58 μM, respectively, and measured between 260 and 180 nm at 1 s per point with 0.1 mm pathlength quartz cuvettes. Initial data processing was achieved with ProData Viewer software v:4.4.2.0, and secondary structure analysis was done with the BeStSel web server [35] for single spectrum and Protein Data Bank (PDB) ID analysis.

### 2.6. Mass spectrometry

Proteomic studies were conducted at the Analytical Biochemistry and Proteomics Unit of the Institut Pasteur de Montevideo. Briefly, protein bands were excised from a Coomassie-stained gel, destained, reduced with 10 mM dithiothreitol (DTT), and alkylated with iodoacetamide, 50 mM. Subsequently, the proteins were digested overnight with trypsin. The resulting peptides were extracted, concentrated, desalted with Stage-Tips and resuspended in 0.1% formic acid. Samples were then injected into a liquid chromatography–mass spectrometry (LC‑MS) spectrometer (LTQ Velos+ETD, Thermo) and analyzed using the MASCOT search engine.

### 2.7. Mouse antiserum against rNcSRS2-ST and rNcSAG1-ST

Two female BALB/c mice (six weeks old) were immunized intraperitoneally once with 100 μg of purified protein mixed with an equal volume of protein and complete Freund’s adjuvant (1 : 1 ratio). Then, they were immunized twice with purified protein and an equal volume of incomplete Freund’s adjuvant (1 : 1 ratio) at 15 day intervals. Mice were bled 15 days after the final immunization [27]. This protocol was performed for each of the proteins separately, as well as the antisera.

### 2.8. Western blot

*Neospora caninum* tachyzoite protein extracts from the Liverpool and Uru1 strains [36] or purified proteins were loaded into a 12% SDS-PAGE gel with equal loads and transferred to polyvinylidene fluoride (PVDF) membranes (Millipore, MA, USA) together with a visible prestained protein marker (PageRuler™ Prestained Protein Ladder, Thermo Scientific™) after electrophoresis [33]. The membranes were blocked with 5% (w/v) skim milk in PBS for 1 h at 37°C, rinsed with a washing buffer and incubated with anti-NcSAG1 and anti-NcSRS2 in 0.1% Tween-20 in PBS (PBST) for 1 h at 37°C (these antibodies were produced as described below). The blots were washed five times with PBST (0.1% Tween−20), followed by incubation with horseradish peroxidase (HRP)-labelled goat anti-IgG (H+L) (1 : 30 000, Sigma, USA). Finally, enhanced chemiluminescence reagents (SuperSignal™ West Pico PLUS Chemiluminescent Substrate, Thermo Scientific™) were used to observe the reaction bands after a 30 s exposure time.

Western blot analysis was also conducted to assess recombinant proteins' expression, size and integrity after induction, using Strep-Tactin-HRP (IBA) following the manufacturer’s recommendations. Briefly, 20 µl of culture supernatant collected after 48 h post-induction were loaded in reducing SDS-PAGE and transferred to a PVDF membrane, as described above. Membranes were blocked with 3% bovine serum albumin (BSA) and 0.05% Tween-20 in PBS for 2 h at 37°C, and after washing three times with 0.1% (v/v) Tween 20 in PBS, were incubated for 1 h with Strep-Tactin® HRP conjugate 1 : 10 000 and revealed by chemiluminescence as described above. We also evaluated whether antibodies from naturally infected animals could recognize the recombinant proteins. To this end, Western blotting was performed as described above, using pooled sera from dogs, cattle and deer that tested positive for *N. caninum* by commercial ELISA. Additionally, sera from mice experimentally infected with 1 × 10⁶ tachyzoites were included. All sera were used at a 1 : 100 dilution, and detection was carried out using protein G-HRP at a 1 : 10 000 dilution.

### 2.9. Structural modelling with AlphaFold

To generate three-dimensional structures from amino acid sequences, we used AlphaFold [37] in monomer mode, running individual predictions for each protein sequence independently. This approach was chosen to evaluate the intrinsic folding of each polypeptide chain. Our focus in this stage was on the tertiary structure of single chains, aiming to characterize sequence-dependent folding features and local structural motifs. The following parameters were applied during AlphaFold running: msa_mode: MMseqs2 (for multiple sequence alignments), num_models: 5 (five models per sequence), num_recycles: 5 (iterative structure refinement) and model_preset: full_dbs (using comprehensive databases). Custom bash scripts automated the process, managing job queuing, resource allocation and error handling to ensure robust execution. The 219 SRS protein sequences analysed were previously identified through a Protein BLAST analysis of the translated *N. caninum* genome [14]. To address AlphaFold’s tendency to model low-confidence regions as unstructured loops, we trimmed the amino and carboxyl termini of each protein, retaining the globular regions and the coil linker loops connecting them. These linker regions, while inherently unstructured, were included for their potential functional relevance. For the identification of structured regions and the selection of truncation sites, we used MKDSSP (version 3.0) to assign secondary structure elements to each AlphaFold model. The resulting annotations were then processed with custom scripts to systematically analyse the secondary structure content across the models. Based on this analysis, we trimmed poorly structured or disordered regions at the N- and C-termini. This approach ensured that only the well-folded, structurally relevant core of each model was retained for downstream analyses.

### 2.10. Structural comparison with pairwise structure alignment

Once the protein structures of NcSAG1 and NcSRS2 were generated, we compared them with the structure of TgSAG1 (PDB ID: 1KZQ) using the web server of Pairwise Structure Alignment [38] with Template Modelling-alignment algorithm (Template Modelling (TM) score). The TM score ranges from 0 to 1; a score greater than 0.5 typically suggests that the two structures have the same fold or topology, meaning they are structurally similar.

### 2.11. Structural comparison with Distance Matrix Alignment

To explore structural similarities and differences within the SRS protein family, we employed Distance Matrix Alignment (DALI) for a detailed structural analysis. Using this approach, we analysed 219 AlphaFold models, using DALI’s robust framework for three-dimensional protein structure comparison [39,40]. DALI conducts pairwise structural comparisons by assessing distance matrices to identify structurally equivalent residues. The method optimizes a scoring function that maximizes the weighted sum of similarities in intramolecular distances between equivalent atom pairs, enabling accurate structural alignment and comparison. We performed all-against-all structure comparison analyses using DALI. This analysis generated a structural similarity dendrogram by systematically comparing each protein structure against all others in the dataset. The resulting dendrogram visually illustrates the structural relationships within the SRS protein family, providing valuable insights into their evolutionary and functional connections.

### 2.12. Structural phylogeny

To investigate the evolutionary relationships within the SRS protein family, we generated amino acid sequence alignments for the 219 sequences mentioned above. These alignments were focused on conserved regions and structural similarities, serving as the basis for subsequent phylogenetic and structural analyses. Gblocks [41] refine the alignment by excluding poorly aligned or ambiguous positions, enhancing the reliability of evolutionary inferences. A maximum likelihood (ML) phylogenetic tree was constructed using MEGA software [42]. The reconstruction employed the JTT+G4+I model of amino acid substitution, selected based on its statistical robustness and suitability for modelling the evolutionary dynamics of these proteins.

### 2.13. Visualization and analysis

Using the similarity matrix obtained from DALI, we constructed a heatmap to visualize the structural clusters of the protein structures. This visualization was created using custom Python scripts, using libraries such as Matplotlib and Seaborn for graphical representation. The heatmap highlights groups of proteins with high structural similarity, facilitating the identification of potential functional or evolutionary relationships. Protein structures were visualized using VMD [43].

### 2.14. Ethics statement

All animal experiments followed international animal care and use guidelines, respecting National Law 18.611 with pre-approved protocols by the Institutional Ethics Committee (CEUA no. 010-17) at the Institut Pasteur de Montevideo.

## 3. Results

### 3.1. Sequence analysis and three-dimensional modelling

**Figure 1.**
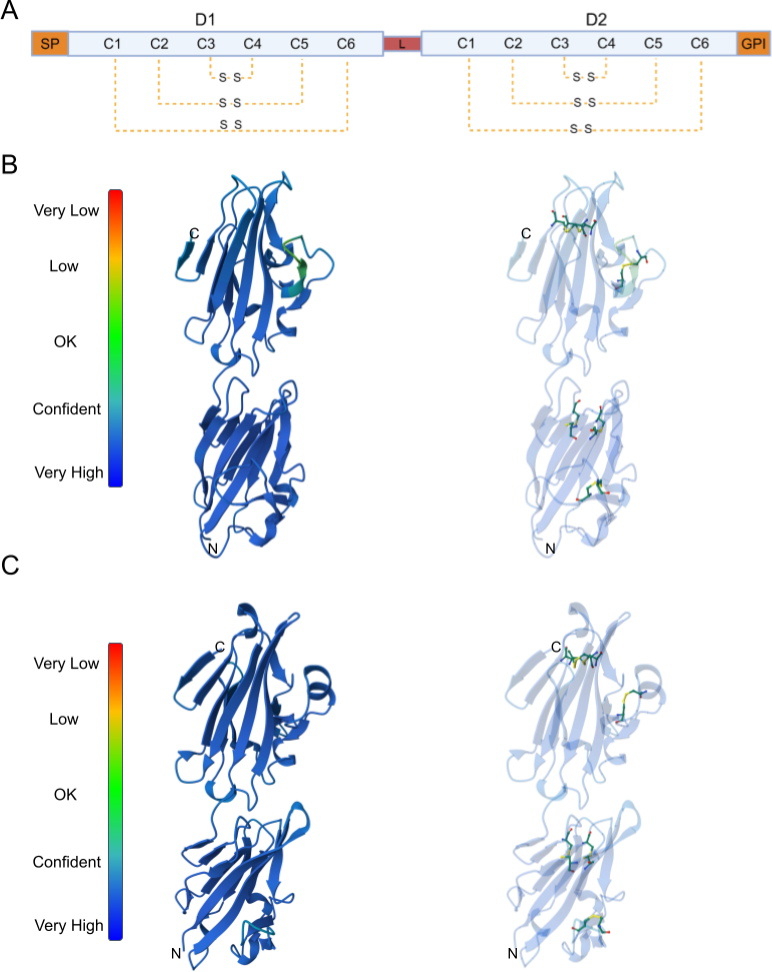
AlphaFold modelling and disulfide bridges in SAG1 and SRS2 of *N. caninum*. (A) Schematic representation of the primary structure, the domains, and the disulfide bridges between cysteines 1 and 6. SP, signal peptide; L, linker; GPI is the anchor site for GPI. (B) AlphaFold model of NcSAG1 (left) and the disulfide bonds (right). (C) AlphaFold model of NcSRS2 (left) and the disulfide bonds (right). For AlphaFold modelling, the SP and GPI anchor regions were removed.

In the literature, NcSAG1 and NcSRS2 are also called p36 (Genbank: AJ005664.1) and p35/p43 (Genbank: AF061249), respectively. Both belong to the SRS protein family and comprise 321 (expected MW: 33,1 kDa) and 401 (expected MW: 38,1 kDa) amino acids, respectively. As members of this family, they share hallmark features, including an N-terminal signal peptide, a C-terminal GPI-anchor, D1 and D2 domains connected by a flexible linker, and six conserved cysteines within each domain (figure 1A). NcSAG1 and NcSRS2 exhibit low overall sequence identity at the amino acid level (electronic supplementary material, table S1). The D1 domains of the two proteins show the highest similarity, with 48% sequence identity, while the D2 domains are more divergent, with only 35% identity. Also, the sequence similarity between the D1 and D2 domains within each protein differs significantly: 24% for NcSAG1 and 38% for NcSRS2.

**Figure 2.**
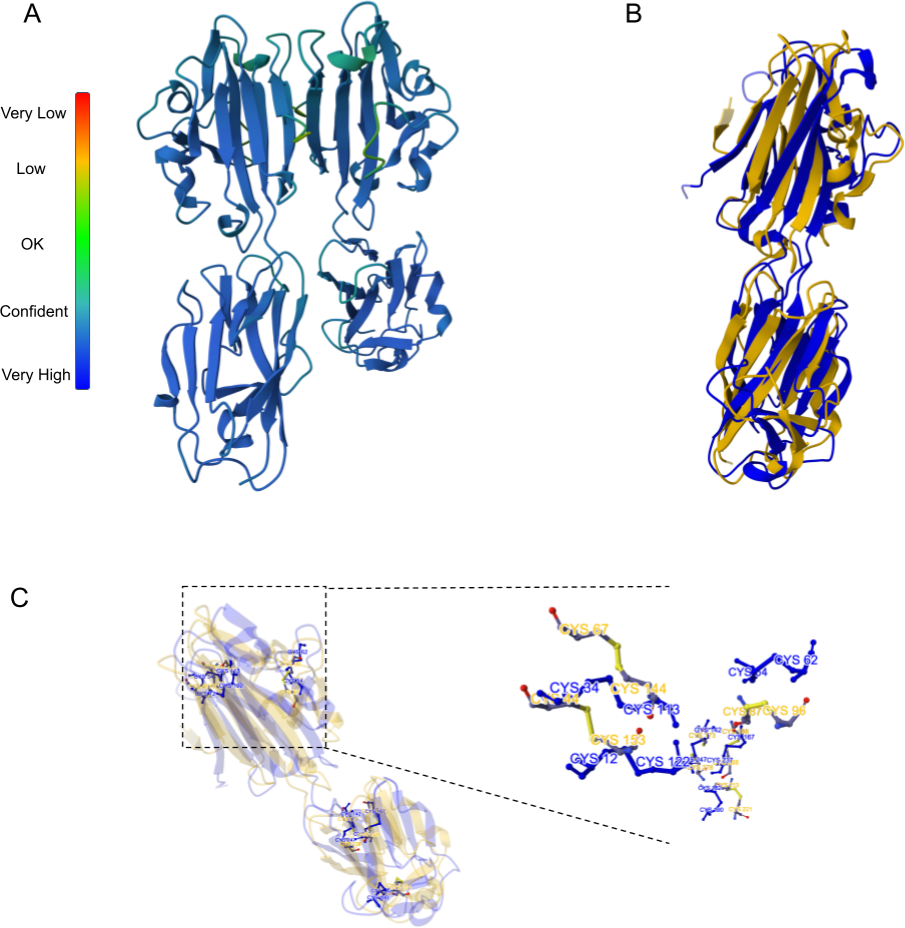
Dimer modelling and structure comparison of NcSAG1 and TgSAG1. (A) AlphaFold model of the NcSAG1 dimer. (B) Structural comparison of NcSAG1 (yellow) with TgSAG1 (PDB 1KZQ; blue). (C) Comparison of disulfide bonds. On the right, the zoomed-in view shows an example of the cysteine residue location.

By using AlphaFold [37], we modelled both proteins, finding the expected D1 and D2 domains, the linker sequence and the conserved cysteines with the pairings 1−6, 2−5 and 3−4 (figure 1B,C). Despite sequence divergence, NcSAG1 and NcSRS2 exhibit significant structural similarity, with a TM score of 0.86 (electronic supplementary material, table S1). The comparison of the D1 domains exhibits a TM score of 0.83, indicating high structural conservation despite their low sequence similarity. By contrast, the D2 domains are more divergent, with a TM score of 0.58. The AlphaFold modelling predicted that NcSAG1 forms a dimer closely resembling the crystallized dimer of TgSAG1, a structural feature potentially relevant to their biological function (figure 2A). We then compared NcSAG1 to the crystallized structure of TgSAG1 through a pairwise structure alignment, obtaining a TM score of 0.61 (figure 2B). Although disulfide bridges in NcSAG1 and NcSRS2 differ in their relative positions to those in TgSAG1 (figure 2C), the overall order of disulfide bonding is conserved (figure 2C).

### 3.2. Recombinant Strep-tagged NcSAG1 and NcSRS2 are expressed as stable and soluble proteins

**Figure 3.**
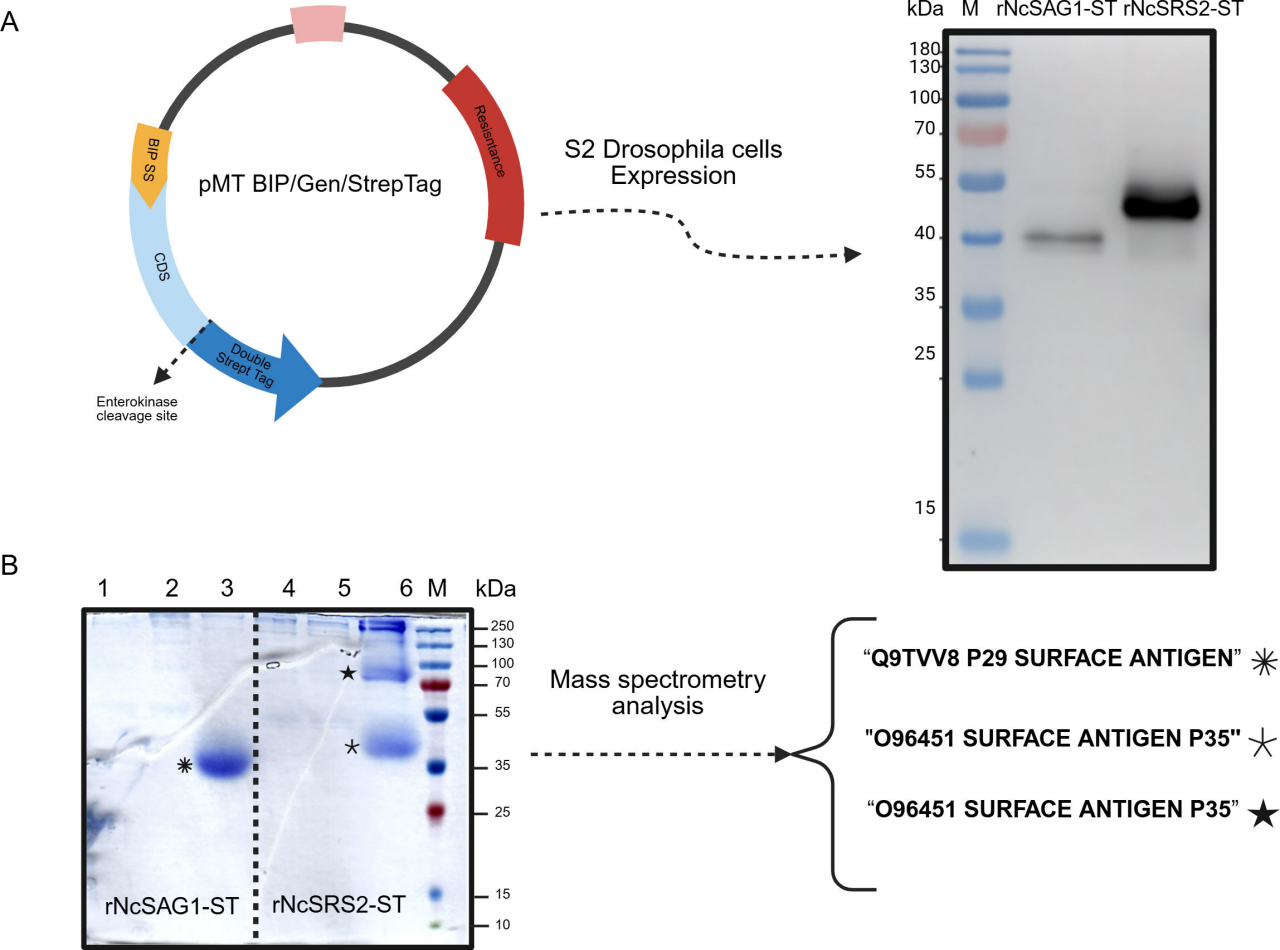
Expression, purification and identification of rNcSAG1-ST and rNcSSR2-ST. (A) The plasmid construction used for protein expression in the *D. melanogaste*r system. Protein expressions were visualized by Western blot in the medium of expression using the Strep-tag. (B) SDS-PAGE analysis of the purification process, where lanes 1 and 4 are total medium fractions, 2 and 5 are the flow-through fractions, and 3 and 6 correspond to the elution fractions from affinity chromatography. Bands stained with Coomassie blue were analysed by mass spectroscopy, identifying the proteins listed on the right.

Both proteins were expressed in S2 cells, as described above. To assess their presence in the soluble fraction, the culture supernatant was collected 48 hours after induction with CdCl_2_ and subjected to AC purification. The expression of rNcSAG1-ST and rNcSRS2-ST was confirmed by Western blot analysis, targeting Strep-tagged proteins. Both proteins appeared as single bands on SDS-PAGE and Western blot, with apparent MWs of approximately 42 and 52 kDa (theoretical MWs 30 and 37 kDa), respectively (figure 3A). Purification by AC allowed us to elute total protein amounts ranging from 2 to 13 mg based on culture scales of 50–450 ml (electronic supplementary material, figure S1). Production yields were 22−65 mg l^−1^ for rNcSAG1-ST and 37−85 mg l^−1^ for rNcSRS2-ST. After affinity purification, both proteins were visible on Coomassie-stained SDS-PAGE (figure 3B). The identity of the bands was confirmed by mass spectrometry: the unique band for rNcSAG1-ST was identified as the p29 surface antigen of *N. caninum*, while both bands observed for rNcSRS2-ST were identified as Ncp35 (figure 3B; electronic supplementary material, table S2). Discrepancies between theoretical and estimated MWs are expected and attributed to post-translational modifications.

### 3.3. Antigenicity and immunogenicity of rNcSAG1-ST and rNcSRS2-ST

**Figure 4.**
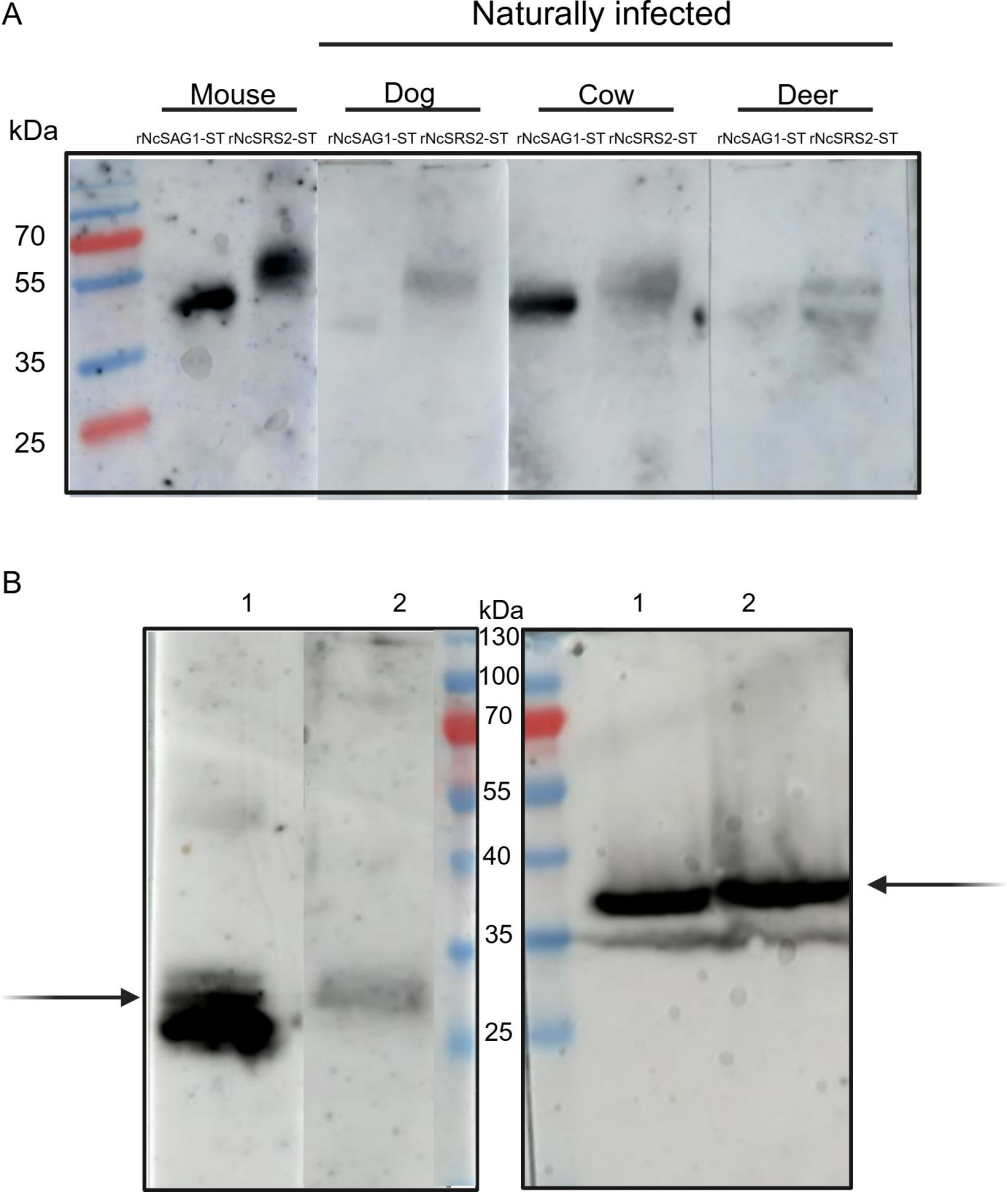
Antigenicity and immunogenicity of rNcSAG1-ST and rNcSRS2-ST. (A) Western blot with antisera from mice immunized with rNcSAG1-ST and rNcSRS2-ST. Lanes 1 and 2 correspond to protein extracts of *N. caninum* from *N. caninum* URU1 and Liverpool strains, respectively. The immune sera recognize native proteins in *N. caninum* extracts. Black arrows indicate the bands corresponding to NcSAG1 and NcSRS2. (B) Western blot analysis of recombinant proteins detected by sera from various infected hosts, including experimentally infected mice and naturally infected cows, dogs and deer.

To evaluate the antigenicity of rNcSAG1-ST and rNcSRS2-ST, we used serum pools from mice, cows, dogs and deer that were positive for neosporosis. Except for the sera from mice, which were derived from experimental infections, all originated from naturally infected animals. They all recognized both proteins, as demonstrated by Western blot (figure 4A). This suggests that they serve as universal antigens, being specifically identified by serum samples from different host species, regardless of whether they were naturally or experimentally infected.

To assess their immunogenicity, we separated total *N. caninum* protein extracts by SDS-PAGE and analysed them via Western blotting using rNcSAG1-ST and rNcSRS2-ST antisera. This approach revealed bands of the expected sizes, with no observed cross-reaction between the antisera (figure 4B). The size differences between recombinant and native proteins were anticipated owing to the addition of biotin in recombinant proteins and variations in post-translational modification profiles.

### 3.4. Stable and dimeric conformation of soluble proteins

**Figure 5.**
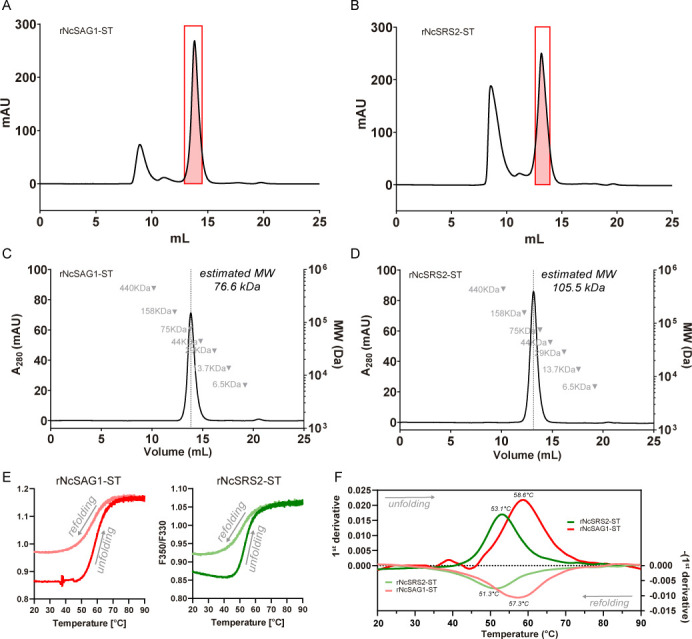
Biochemical and physicochemical characterization of rNcSAG1-ST and rNcSRS2-ST. (A and B) Preparative size exclusion chromatography (SEC) of rNCSAG1-ST and rNcSRS2-ST, respectively, with the main collected peak shadowed in red. (C and D) Analytical SEC of the main fractions of rNcSAG1-ST and rNcSRS2-ST obtained in (A) and (B), respectively. The molecular weight (MW) calibration curve is included with values in grey, and the estimated MW of each protein is shown in kilodaltons (kDa) next to each peak. (E) Nano differential scanning fluorimetry showing changes in intrinsic fluorescence of rNcSAG1-ST (red) and rNcSRS2-ST (green) upon heating and cooling (shown in bright and pale colours, respectively, accompanied by grey arrows indicating the direction of each transition). (F) The first derivative of the curves obtained in (E), shows the melting temperature (*T*_M_) as the peak of each transition at a temperature of Celsius degrees. ST means that the protein contains a C-terminal double Strep-tag.

**Figure 6.**
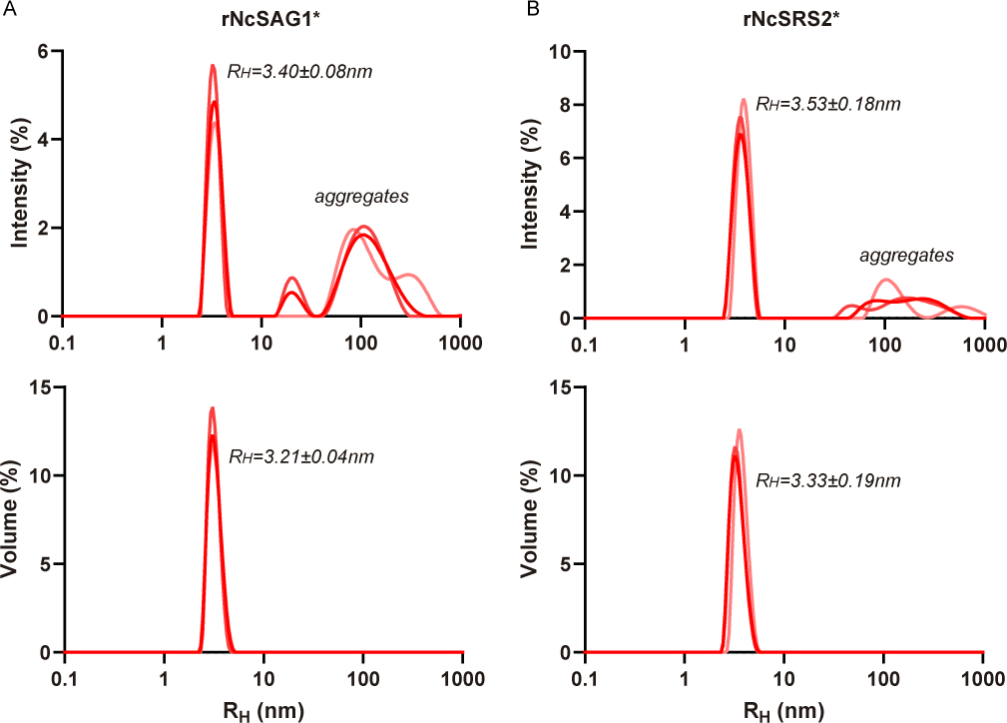
Evaluation of rNcSAG1* and rNcSRS2* behaviour in solution. Hydrodynamic radius (*R*_H_) of rNcSAG1* and rNcSRS2*, respectively, measured by DLS after treatment with bovine enterokinase to remove the Strep-tag. Distributions calculated by intensity (upper panels) show a main peak at 3.53 and 3.40 nm, respectively, accompanied by high molecular weight aggregates, which are not detected in distributions measured by volume (lower panels). Asterisk suffixes represent that the Strep-tag was removed.

The inclusion of an extra purification step by SEC enabled us to obtain several fractions with different oligomerization states. Within each purification batch, we isolated both proteins as a main peak preceded by high MW aggregates (figure 5A,B). When these main fractions were isolated and re-injected in analytical SEC, rNcSAG1-ST and rNcSRS2-ST eluted as unique peaks with estimated MWs of 105.5 and 76.6 kDa, suggesting that both proteins are obtained as stable dimers (figure 5C,D). The thermostability of rNcSAG1-ST and rNcSRS2-ST was assessed by nanoDSF, showing thermal unfolding with *T*_M_ above 50°C (*T*_M_ = 58.6°C for rNcSAG1-ST and *T*_M_ = 53.1°C for rNcSRS2-ST). Additionally, both proteins showed reversibility with no signs of aggregation upon heating to 90°C (figure 5E,F). Both dimeric rNcSAG1-ST and rNcSRS2-ST were purified by preparative SEC and treated overnight with bovine enterokinase to remove the Strep-tag. Subsequently, cleaved proteins (rNcSAG1* and rNcSRS2*) were re-injected in a preparative SEC, showing a unique peak with a slight shift in exclusion volume towards smaller sizes, as a consequence of the Strep-tag removal (electronic supplementary material, figure S2). The main peak was collected and concentrated 10-fold to obtain batches of cleaved versions of dimeric proteins at 1 mg ml^−1^. Batches of both dimeric rNcSAG1* and rNcSRS2* were analysed by DLS to evaluate their behaviour in solution after Strep-tag removal and sample concentration. Both proteins showed size distributions by intensity, containing a main population with an average hydrodynamic radius (*R*_H_) of 3.40 nm for rNcSAG1* and 3.53 nm for rNcSRS2*, and high-sized aggregates, which are negligible, as observed in size distributions by volume (figure 6).

### 3.5. Circular dichroism

**Figure 7.**
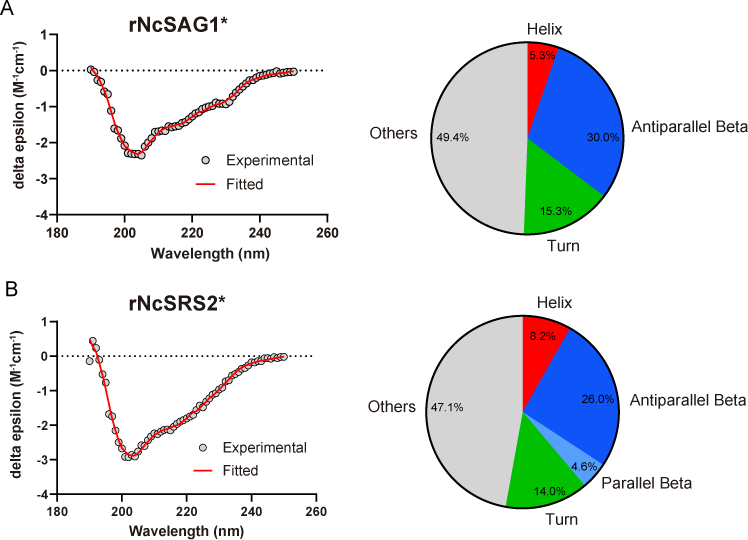
Secondary structural analysis by circular dichroism. (A and B) Spectral data and deconvolution interpretation for rNcSAG1* and rNcSRS2*, respectively.

Far-UV CD spectra of rNcSAG1* and rNcSRS2* were found to be very similar, suggesting that both proteins have a comparable secondary structure composition. Deconvolution of spectra using BeStSel allowed us to identify a secondary structure content of approximately 30% β-strands (mainly relaxed and right-twisted antiparallel β-strands) followed by 14%–15.3% of turns and 8.2%–5.3% of α-helices (mainly distorted α-helices) as seen in figure 7. To validate our structural interpretation of CD spectra using BeStSel, we carried out an orthogonal approach based on the PDB structures of related proteins such as TgSAG1, TgBSR4 and TgSporoSAG from *T. gondii*, obtaining quite similar secondary structure compositions for all of them (electronic supplementary material, figure S3).

### 3.6. Comparative structural analysis

**Figure 8.**
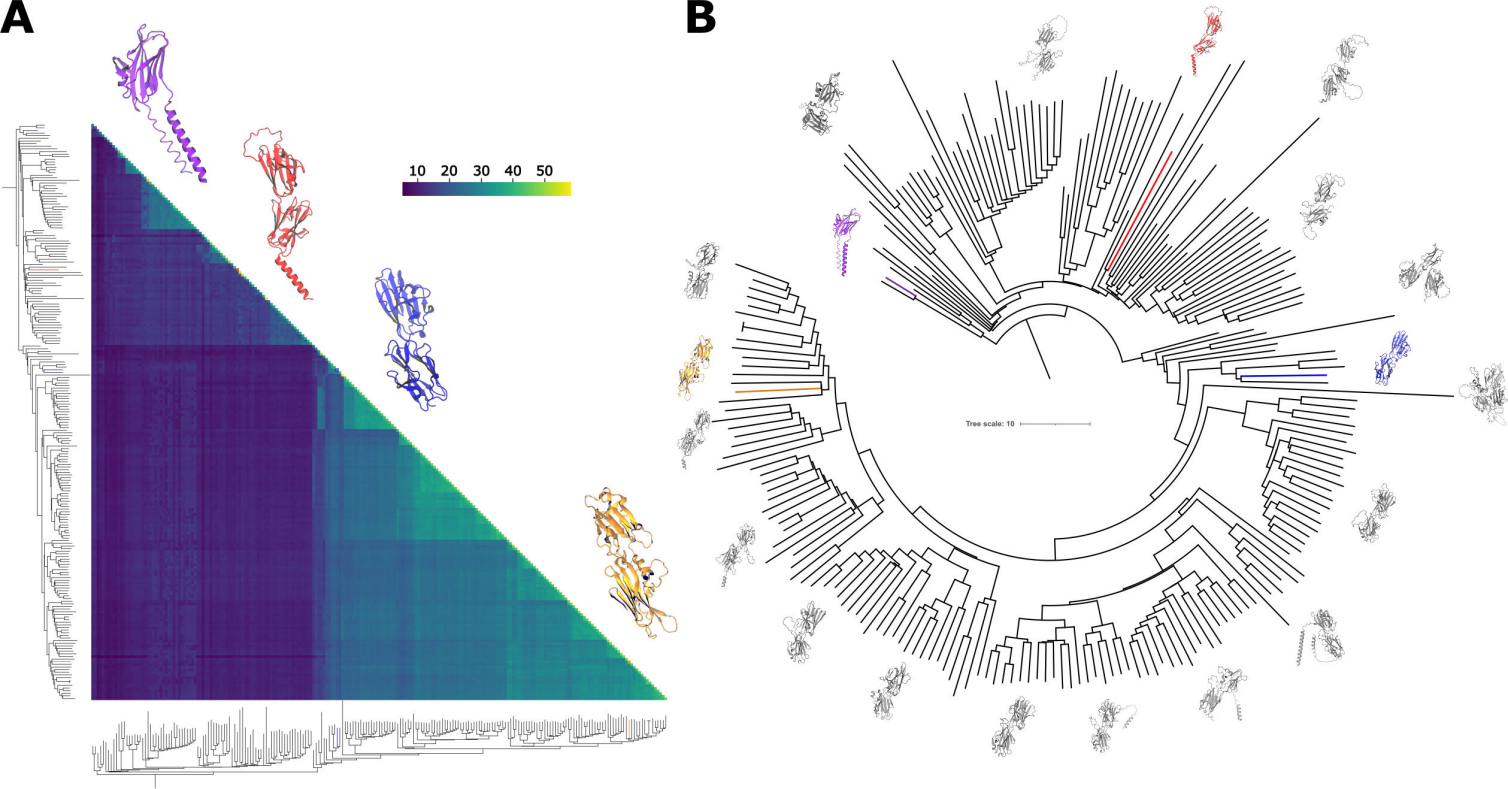
Comprehensive analysis of structural similarity through heatmap and dendrogram. (A) The heatmap visualizes the structural similarity among proteins using a colour gradient that ranges from blue to yellow, indicating low to high similarity, respectively. This heatmap displays the lower triangular region of the similarity matrix. Along the axes of the heatmap, a dendrogram is plotted to illustrate the hierarchical clustering of these structures. Key structures of interest relevant to this study are distinctly highlighted: Ncaninum000533300 (SAG4) is marked in purple, Ncaninum000104000 (an orthologue of SporoSAG) in red, Ncaninum000061800 (SAG1) in blue and Ncaninum000678300 (BSR4) in yellow. (B) The star-shaped dendrogram, derived from the structural similarity matrix, effectively depicts the structural relationships and diversity among the analysed proteins. Each clade within the dendrogram is represented by a unique, randomly selected structure, coloured in silver, to exemplify the variation and commonalities within the clusters. This representation underscores the breadth of structural diversity and aids in identifying significant similarities and differences within the dataset. The relevant protein structures highlighted in this study are coloured as in panel (A).

From a protein BLAST analysis of the translated *N. caninum* genome, 219 sequences belonging to the SRS family were selected (electronic supplementary material, table S3; [14]). These sequences retain key SAG domain features, such as conserved cysteines, prolines and the signature tryptophan. To compare these proteins, we employed AlphaFold for structure prediction and DALI for structural comparison, enabling a systematic exploration of their structural features and relationships. DALI generated a similarity matrix for the 219 predicted protein structures, quantified by *Z*-scores that indicate the degree of structural similarity between protein pairs. Higher *Z*-scores signify greater similarity. The triangular heatmap (figure 8A) illustrates this matrix, with yellow indicating high similarity and purple denoting divergence. Hierarchical clustering along the axes groups proteins with similar structural characteristics, revealing distinct clusters within the SRS family. Representative structures from key clusters are highlighted, illustrating conserved and variable motifs. Some clusters belonging to previously biologically characterized proteins are marked in red, blue, orange and purple, which include Ncaninum_LIV_000104000 (an orthologue of SporoSAG), Ncaninum_LIV_000061800 (NcSAG1), Ncaninum_LIV_000678300 (NcBSR4) and Ncaninum_LIV_000533300 (NcSAG4), respectively (figure 8A,B).

To evaluate the robustness of our methodology, we compared the ML phylogenetic tree, based on sequence data processed with Gblocks, with the structure-based tree derived from DALI (electronic supplementary material, figure S5). Both trees highlighted the major clusters, demonstrating a strong correlation between sequence- and structure-based phylogenetic analyses. While congruences reinforced the reliability of our approach, some discrepancies between sequence and structural evolution suggest instances of structural divergence not mirrored in sequence data. These findings underscore the complementarity of sequence- and structure-based methodologies, offering a comprehensive understanding of the SRS protein family in *N. caninum*.

These results establish a framework for further investigation into the roles and interactions of SRS proteins, bridging structural diversity and evolutionary patterns to their biological significance.

## 4. Discussion

This study reports the successful expression, purification and characterization of NcSAG1 and NcSRS2 proteins. The *D. melanogaster* S2 expression system was chosen since it has proven advantageous, enabling efficient production of secreted proteins while simplifying purification and scalability [34,44]. In addition, it could address challenges often encountered with prokaryotic systems, including insolubility and low expression levels, and preserve the disulfide bridges, which play a crucial role in the structural integrity of these proteins, as described for *T. gondii* SRS proteins [17–19]. Moreover, since they are glycoproteins, incorporating post-translational modifications is necessary. It should be noted that the glycosylation pattern in S2 cells may not fully replicate that of *N. caninum*, which could influence epitope recognition and modulate the immune response. Both rNcSAG1-ST and rNcSRS2-ST were expressed as soluble proteins, with production yields comparable to the most efficient prokaryotic systems, making the S2 system highly recommended for expressing proteins of eukaryotic origin. The stable and soluble production of rNcSAG1-ST and rNcSRS2-ST enabled us to complement the structural studies with physicochemical characterization, as discussed later.

Both proteins exhibit hallmark features of the SRS family, including an N-terminal signal peptide, a C-terminal GPI-anchor, D1 and D2 domains connected by a flexible linker, and 12 conserved cysteines forming characteristic disulfide bonds. Despite their low overall sequence identity, NcSAG1 and NcSRS2 share significant structural similarities, as evidenced by their TM scores of 0.86 for full-length proteins and 0.83 for the D1 domains. These findings suggest that structural conservation may be critical in maintaining their functional integrity. The D1 domains, which exhibit higher sequence identity than the D2 domains, may contribute to a conserved core function, whereas the more divergent D2 domains could support specialized or distinct roles. The AlphaFold-predicted structures highlight the importance of disulfide bonding patterns in stabilizing the architecture of these proteins. While the relative positions of disulfide bridges differ between NcSAG1, NcSRS2 and the structurally resolved TgSAG1, the overall disulfide bonding order remains conserved. The more stable predicted structures are dimeric, and its resemblance to the crystallized dimer of TgSAG1 suggests that this dimeric arrangement may be essential for biological functions such as host cell attachment or immune evasion, as previously reported for other SRS proteins. This result is supported here by experimental assays, as discussed below. The moderate TM score (0.61) obtained from the structural comparison between NcSAG1 and TgSAG1 underscores both conserved and divergent aspects of their structures, potentially reflecting adaptations to different host environments or immune pressures.

The information obtained from structure prediction was confirmed through biochemical and biophysical studies. In the first purification step, both proteins were eluted with a main peak consistent with the formation of a dimer. Including an additional SEC step significantly enhanced the purity and resolution of oligomeric states, enabling the isolation of rNcSAG1-ST and rNcSRS2-ST as stable dimers. This stability is further corroborated by the high *T*_M_ observed in nanoDSF assays (58.6°C for rNcSAG1-ST and 53.1°C for rNcSRS2-ST), which are notable for recombinant proteins. The ability of these proteins to refold after heating to 90°C without aggregation suggests robust structural integrity, making them suitable for diverse experimental studies. We can suppose that the dimer is the functional unit in both cases, with direct implications for future studies on potential ligands and inhibitors. In addition, the thermostability may reflect evolutionary adaptations that ensure functionality in varying environments, a feature worth exploring in the context of their native biological roles.

The far-UV CD analysis of rNcSAG1* and rNcSRS2* provides valuable insights into their secondary structure and strengthens the robustness of the AlphaFold modelling. The close agreement between the CD-based secondary structure estimates for rNcSAG1* and rNcSRS2* and those derived from TgSAG1, TgBSR4 and TgSporoSAG suggests a conserved architectural motif within this protein family. This conservation underscores the evolutionary pressure to maintain structural elements essential for function, such as ligand binding or host-pathogen interactions. The predominance of relaxed and right-twisted antiparallel β-strands indicates a structural preference that may be key to their stability and interactions with other molecules. Additionally, the presence of distorted α-helices and turns reflects structural versatility, which could play a role in facilitating dynamic conformational changes required for their functions. These results suggest that the β-strands constitute the backbone of this protein family, providing stability and conservation, enhanced by the disulfide bridges between the six highly conserved cysteines for each domain; meanwhile, the α-helices and turns act as sources of variability under evolutionary pressure, enabling functional and structural diversity.

The biochemical and physicochemical analyses suggest that the recombinant proteins are of high quality and suitable for developing advanced diagnostic techniques and vaccines. Proteins that accurately mimic native folding and post-translational modifications are expected to enhance immune system recognition, which is critical for effective diagnostic assays and vaccine development. Previous vaccine development efforts have used protein fragments or insoluble forms, limiting antigenic recognition to specific sequences [26,28,45]. The absence of NcSAG1 reduces parasite infectivity, suggesting that antibody-mediated neutralization of this protein could significantly decrease infective capacity. However, redundancy within the protein family may necessitate the inclusion of multiple antigens for full inhibition [30]. A recent study with recombinant NcSAG1 expressed in *Escherichia coli* demonstrated protective efficacy in cows, eliciting strong humoral responses and activation of peripheral blood mononuclear cells (PBMCs) [46]. Incorporating NcSAG1 and NcSRS2 in forms closer to their native structures could further enhance these results. In this sense, we generated murine antisera against both recombinant proteins, and they successfully recognized *N. caninum* lysates, including reference and local strains. Additionally, sera from infected animals recognized these proteins, confirming specificity. The absence of cross-reactivity among antibodies targeting different recombinant proteins also highlights their diagnostic potential. Combining multiple antigens could improve serological test sensitivity and specificity [47].

The strong correlation between structural modelling and CD spectroscopy data enabled the construction of the first structure-based phylogenetic tree for the SRS protein family. This integrative analysis, based on AlphaFold-generated monomeric models and DALI-based structural comparisons, provides a comprehensive overview of the family’s structural landscape and evolutionary relationships. It is important to emphasize that all computational analyses, including structure prediction, clustering and tree reconstruction, were conducted using monomeric forms of the proteins, and the results should be interpreted as structural hypotheses rather than definitive conclusions.

Our results revealed distinct structural clades that align with known sequence-based groupings and support hypotheses regarding potential functional specialization. In *T. gondii*, SRS proteins cluster into two well-characterized groups (SAG1 and SAG2A) [15,48]; a similar pattern is observed in *N. caninum*, where NcSAG1 and NcSRS2 form a distinct subclade within the SAG1-like group, possibly reflecting species-specific adaptations. Unlike previous studies reporting stage-specific clustering for *T. gondii*, our structural tree does not segregate proteins by life stage, suggesting that structural similarity might relate more to functional features than to temporal expression, though this remains a working hypothesis.

The subset of proteins expressed in this study falls within the same clade and groups coherently with sequence-based classifications. Secondary structure content derived from CD spectroscopy exhibited strong agreement with the structural features predicted by AlphaFold, supporting the validity of the models.

Representative structures shown in figure 8A span from approximately 180–420 amino acids in length. Shorter models typically feature a single β-sandwich domain, which may correspond to naturally occurring isoforms or truncations potentially derived from alternative splicing. The presence of a single domain raises the possibility that this structural unit alone could fulfill essential biological functions—an intriguing hypothesis that warrants future experimental validation. Additionally, several models display extended α-helices projecting from the core fold. Based on their orientation and hydrophobic character, these helices may represent membrane-anchoring regions or transmembrane domains. While detailed biophysical or topological analyses were beyond the scope of this work, these features merit further investigation to fully elucidate their functional relevance.

In conclusion, our results and generated tools provide a foundation for further exploration of these proteins' biological roles. The successful expression of soluble, stable proteins positions this system as a promising method for producing antigens for both diagnostic and vaccine applications, offering new avenues for improving the management and understanding of neosporosis.

## Data Availability

Data can be accessed online [49]. Supplementary material available online [50].
